# Non-Oliguric IRIS Grade V Acute Kidney Injury Associated with Suspected Leptospirosis in an Urban Dog Unvaccinated Against Leptospirosis from the Republic of Korea: A Case Report

**DOI:** 10.3390/vetsci13090982

**Published:** 2026-09-17

**Authors:** Taehyun Roh, Kunho Song

**Affiliations:** 1 Department of Veterinary Internal Medicine, College of Veterinary Medicine, Chungnam National University, Daejeon 34134, Republic of Korea; freegith@gmail.com; 2 Soop Animal Medical Center, Daejeon 35233, Republic of Korea

**Keywords:** leptospirosis, acute kidney injury, non-oliguric, real-time polymerase chain reaction, LipL32, zoonosis

## Abstract

Leptospirosis is a zoonotic disease that can cause severe acute kidney injury (AKI) in dogs. A three-year-old intact male Border Collie, unvaccinated against leptospirosis, with a history of exposure to urban stagnant water presented with acute vomiting, diarrhea, and moderate dehydration. Initial evaluation revealed extreme azotemia (blood urea nitrogen, 175.8 mg/dL; creatinine, 11.10 mg/dL) and hyperphosphatemia (13.6 mg/dL), consistent with International Renal Interest Society (IRIS) AKI Grade V; urine output remained within the non-oliguric range (2.15–3.0 mL/kg/h) throughout the catheterized period (Day 0–4). Clinical and imaging findings supported acute intrinsic renal injury over chronic kidney disease. A multiplex real-time PCR targeting the *Leptospira LipL32* gene was positive in urine (Ct 31.10), providing molecular evidence supporting active infection or shedding of pathogenic *Leptospira* spp., without identifying the infecting serovar. Empirical antimicrobial therapy targeting suspected leptospirosis, together with intravenous fluid therapy, was initiated on clinical suspicion prior to the PCR result. Renal and inflammatory biomarkers improved progressively over 22 days without extracorporeal kidney support; the patient’s non-oliguric status, hemodynamic stability, and objective clinical improvement supported this management approach. This report documents, in detail, the clinicopathological recovery trajectory of a dog with non-oliguric IRIS Grade V AKI associated with suspected leptospirosis, information that may assist clinicians in recognizing and managing similarly severe presentations without assuming that dialysis is always required.

## Simple Summary

Leptospirosis is a bacterial disease that can spread from animals to humans and can cause sudden, severe kidney injury in dogs. This case report describes a three-year-old Border Collie, unvaccinated against leptospirosis, with a history of walking along an urban riverbank, that developed extremely severe acute kidney injury together with molecular evidence supporting infection with pathogenic *Leptospira* bacteria. Despite the severity of the kidney injury, the dog continued to produce urine during the period it was monitored (the first five days of hospitalization) rather than producing very little or none—a pattern called non-oliguric kidney injury. Treatment combined antibiotics with intravenous fluids, and the dog’s kidney values improved steadily over three weeks without the need for dialysis. This report documents, in detail, the day-by-day recovery of a dog with this severe but non-oliguric form of kidney injury, information that may help veterinarians recognize similar cases and make informed decisions about monitoring and treatment intensity.

## 1. Introduction

Leptospirosis is caused by infection with pathogenic *Leptospira* species and is one of the most widespread zoonotic diseases globally [1]. In dogs and humans, the disease ranges from subclinical infection to fatal multiorgan failure involving acute kidney injury (AKI), hepatic failure, and hemorrhagic disorders [1,2]. In the Republic of Korea, human leptospirosis has historically been associated with rural, autumn-season exposure to rodent reservoirs [3,4], and recent serological surveys have identified leptospiral antibodies in domestic companion dogs, raising concerns regarding zoonotic risk from this population [5]. Canine leptospirosis in urban settings has also been increasingly recognized internationally [6,7]; however, urban exposure alone does not, by itself, distinguish an individual case from previously published reports.

Severe leptospirosis-associated AKI, classified as IRIS Grade V, generally carries a guarded prognosis, and referral for extracorporeal kidney support (EKST) is frequently recommended [8,9]. This report describes a dog unvaccinated against leptospirosis with IRIS Grade V AKI and suspected leptospirosis that remained non-oliguric throughout the catheterized period (Day 0–4) and achieved favorable clinical recovery with conventional medical management. We present a detailed, serial clinicopathological account of this recovery to provide clinicians with reference data for similarly severe, non-oliguric presentations, and to reinforce the importance of early clinical recognition, the supportive (rather than confirmatory) role of molecular testing, and preventive vaccination.

## 2. Case Presentation

### 2.1. Signalment, History, and Presentation

A 3-year-old intact male Border Collie (17.3 kg) presented with a 4–5-day history of vomiting (approximately four episodes per day) and diarrhea (approximately two episodes per day), accompanied by progressive lethargy. Changes in water intake or urination prior to presentation were not specifically noted by the owner. The dog had a history of walking along urban ecological river trails in Daejeon, Republic of Korea, and of playing in and drinking from stagnant water. Core vaccinations (Distemper, Hepatitis, Parvovirus, Parainfluenza; DHPP) were up to date, but the dog had never received a *Leptospira*-containing vaccine and was therefore considered unvaccinated against leptospirosis. On presentation (Day 0), physical examination revealed moderate dehydration (7–8%), characterized by dry mucous membranes and prolonged skin turgor; body temperature, heart rate, and respiratory rate were within normal limits, and there was no history of chronic weight loss.

### 2.2. Clinicopathological Findings

**Figure 1:**
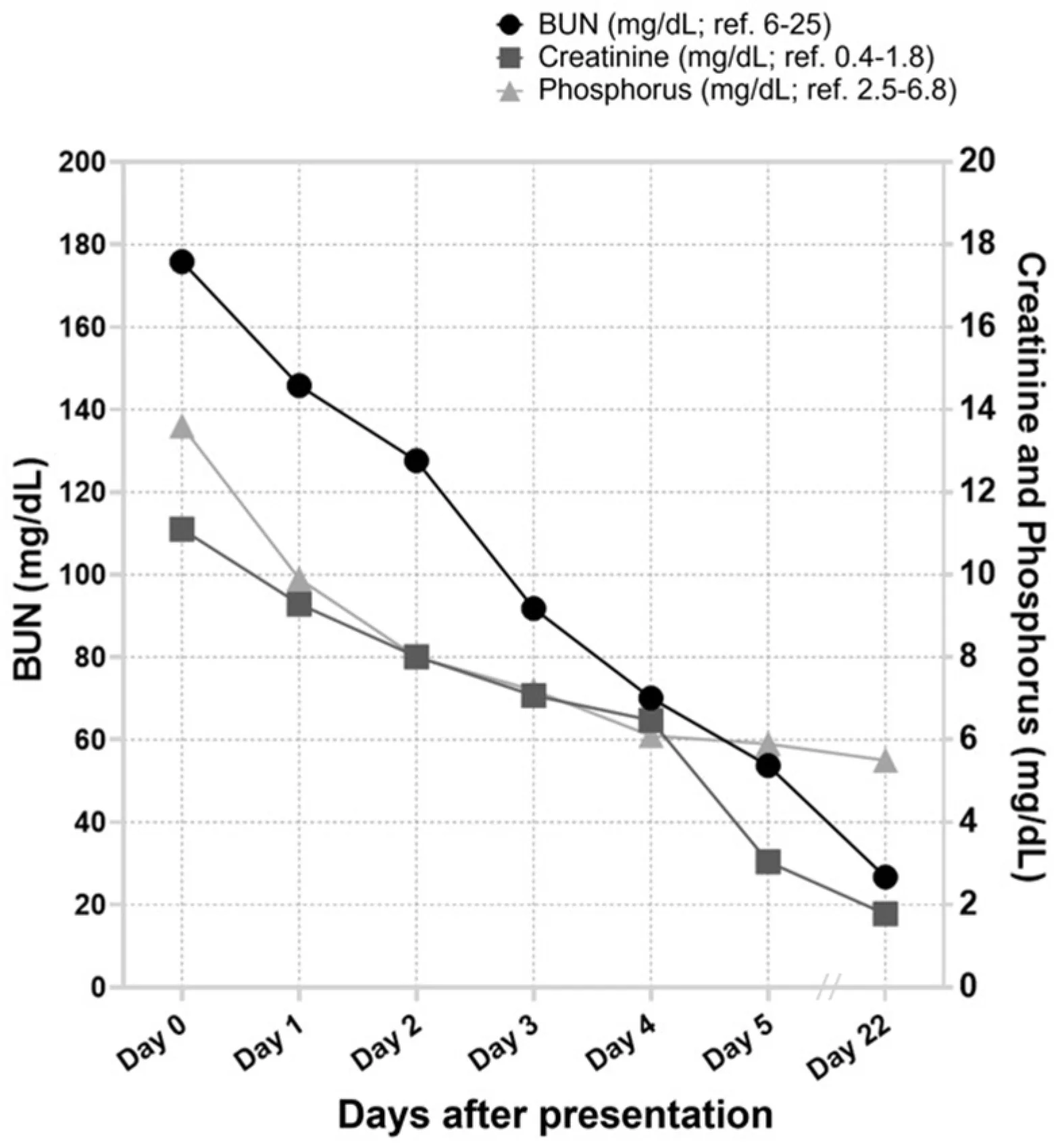
Longitudinal changes in major renal parameters (blood urea nitrogen [BUN], serum creatinine, and inorganic phosphorus) from admission (Day 0) to follow-up (Day 22). Reference intervals for each parameter are indicated in the legend (BUN, 6–25 mg/dL; creatinine, 0.4–1.8 mg/dL; phosphorus, 2.5–6.8 mg/dL).

**Figure 2:**
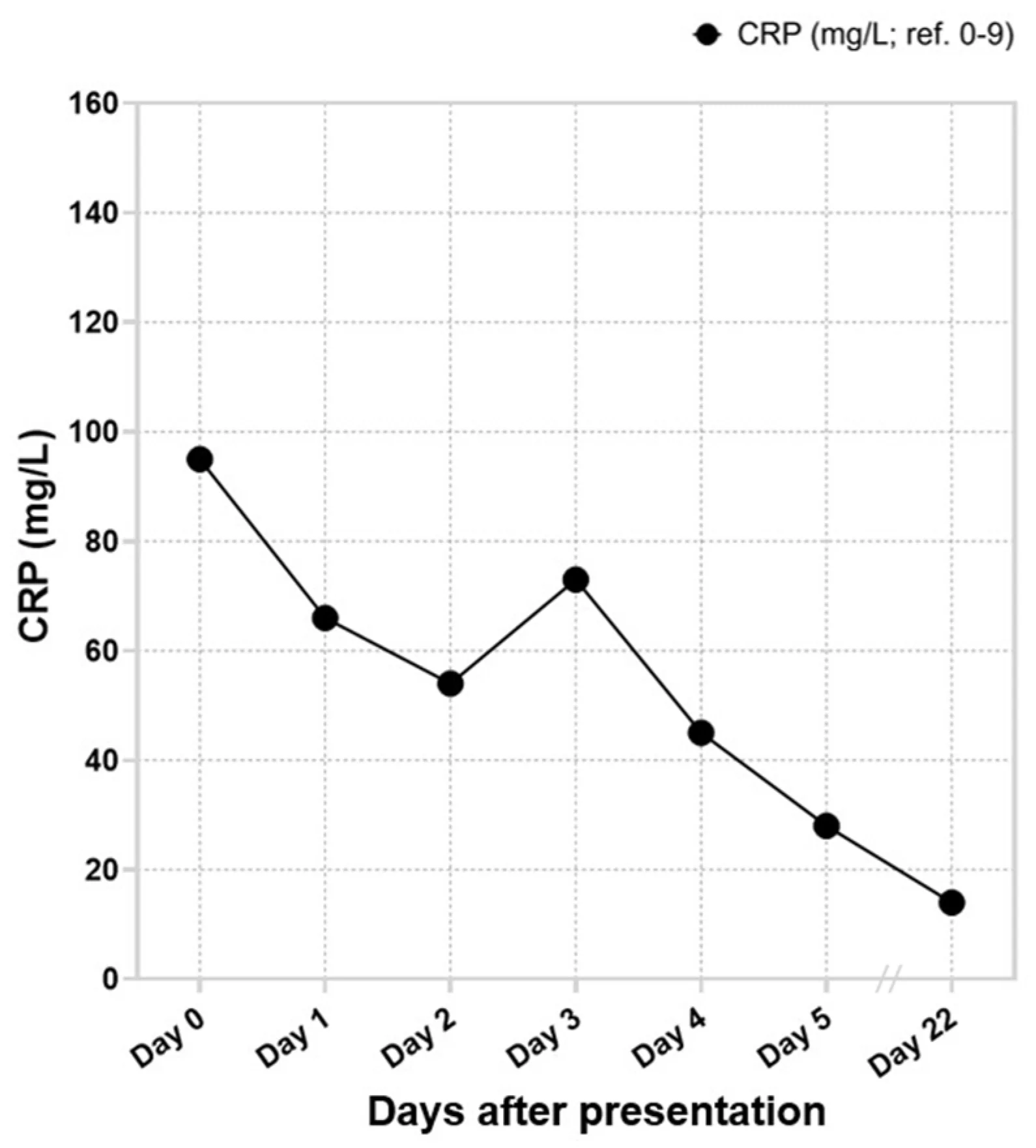
Longitudinal changes in the systemic inflammatory marker canine-specific C-reactive protein (c-CRP) from admission (Day 0) to follow-up (Day 22). The reference interval (0–9 mg/L) is indicated in the legend.

A comprehensive initial laboratory panel (complete blood count, serum biochemistry, electrolytes, venous blood gas analysis, and urinalysis) was performed immediately; results are summarized in Table 1. Serum biochemistry revealed extreme azotemia (BUN 175.8 mg/dL; creatinine 11.1 mg/dL; Figure 1) and hyperphosphatemia (13.6 mg/dL), consistent with IRIS AKI Grade V, together with pronounced systemic inflammation (c-CRP 95 mg/L; Figure 2).

Serum albumin and total protein were within reference intervals. Although normal values do not definitively exclude chronic kidney disease, these findings—together with the acute clinical history, bilateral renal enlargement, and preserved corticomedullary architecture on ultrasonography—were more consistent with acute intrinsic renal injury than with chronic protein-losing nephropathy; acute-on-chronic injury (i.e., a new acute insult superimposed on pre-existing, previously undiagnosed chronic kidney disease) could not be entirely excluded without prior renal data, although the rapid, marked decline in azotemia made significant pre-existing chronic kidney disease unlikely.

Venous blood gas and electrolyte values are summarized in Table 1. Sodium was mildly elevated (154 mmol/L; reference interval, 139–151 mmol/L), consistent with the patient’s dehydration. This finding was not considered clinically significant and was expected to resolve with standard fluid therapy. Potassium was mildly decreased and the anion gap was mildly elevated; all other electrolyte and blood gas values were within reference intervals. Hematocrit decreased from 46.0% (Day 0) to 35.5% (Day 1) following intravenous fluid resuscitation, most consistent with hemodilution; reticulocyte indices were consistent with a non-regenerative response, as expected within this acute time frame.

Urinalysis (Table 1) revealed inappropriately dilute urine (specific gravity 1.015) given the degree of dehydration, consistent with impaired renal concentrating ability due to intrinsic tubular damage rather than a purely prerenal process. Mild proteinuria (urine protein-to-creatinine ratio 0.79) and renal glucosuria despite normoglycemia were also present, findings consistent with proximal tubular dysfunction. Urine culture was not performed, and molecular testing was used instead (Section 2.4); the absence of bacteria on sediment examination does not exclude urinary tract infection or pyelonephritis.

### 2.3. Diagnostic Imaging

**Figure 3:**
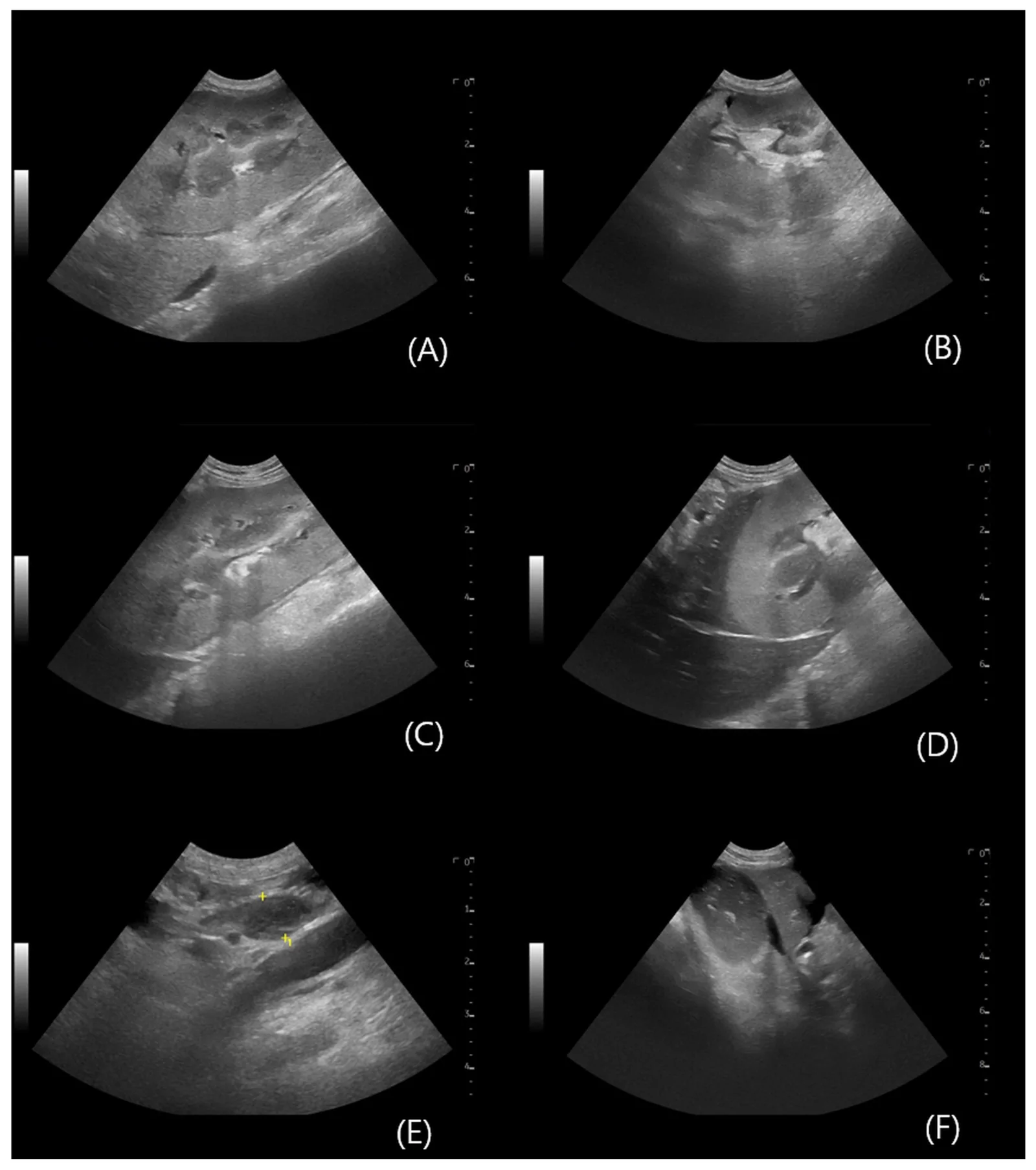
Abdominal ultrasonographic findings on presentation. (**A**,**B**) Sagittal and transverse views of the left kidney, respectively. (**C**,**D**) Sagittal and transverse views of the right kidney, respectively. Both kidneys demonstrate a diffuse increase in cortical echogenicity. (**E**) An enlarged mesenteric lymph node indicating reactive lymphadenopathy; yellow crosses indicate caliper markers used to measure the lymph node. (**F**) A small amount of anechoic perirenal fluid accumulation. The grayscale bar adjacent to each image represents the standard brightness reference scale of the ultrasound system display and carries no additional diagnostic significance.

Abdominal ultrasonography (GE LOGIQ Fortis; GE HealthCare, Chicago, IL, USA; C3–10 microconvex probe, 3.0–10.0 MHz, imaging frequency 10.0 MHz) revealed symmetrically enlarged kidneys with smooth contours, diffuse increased cortical echogenicity, and preserved corticomedullary differentiation, without evidence of chronic architectural change; mild reactive mesenteric lymphadenopathy and a small volume of perirenal fluid were also noted (Figure 3). Because the kidneys could not be captured in their entirety within a single ultrasonographic field of view using the available probe, renal length and the renal-to-aortic diameter ratio could not be obtained sonographically. The liver and biliary tract were also evaluated; hepatic parenchymal echogenicity, gallbladder wall thickness, and biliary duct diameter were unremarkable, with no sonographic findings suggestive of hepatitis or biliary tract disease.

**Figure 4:**
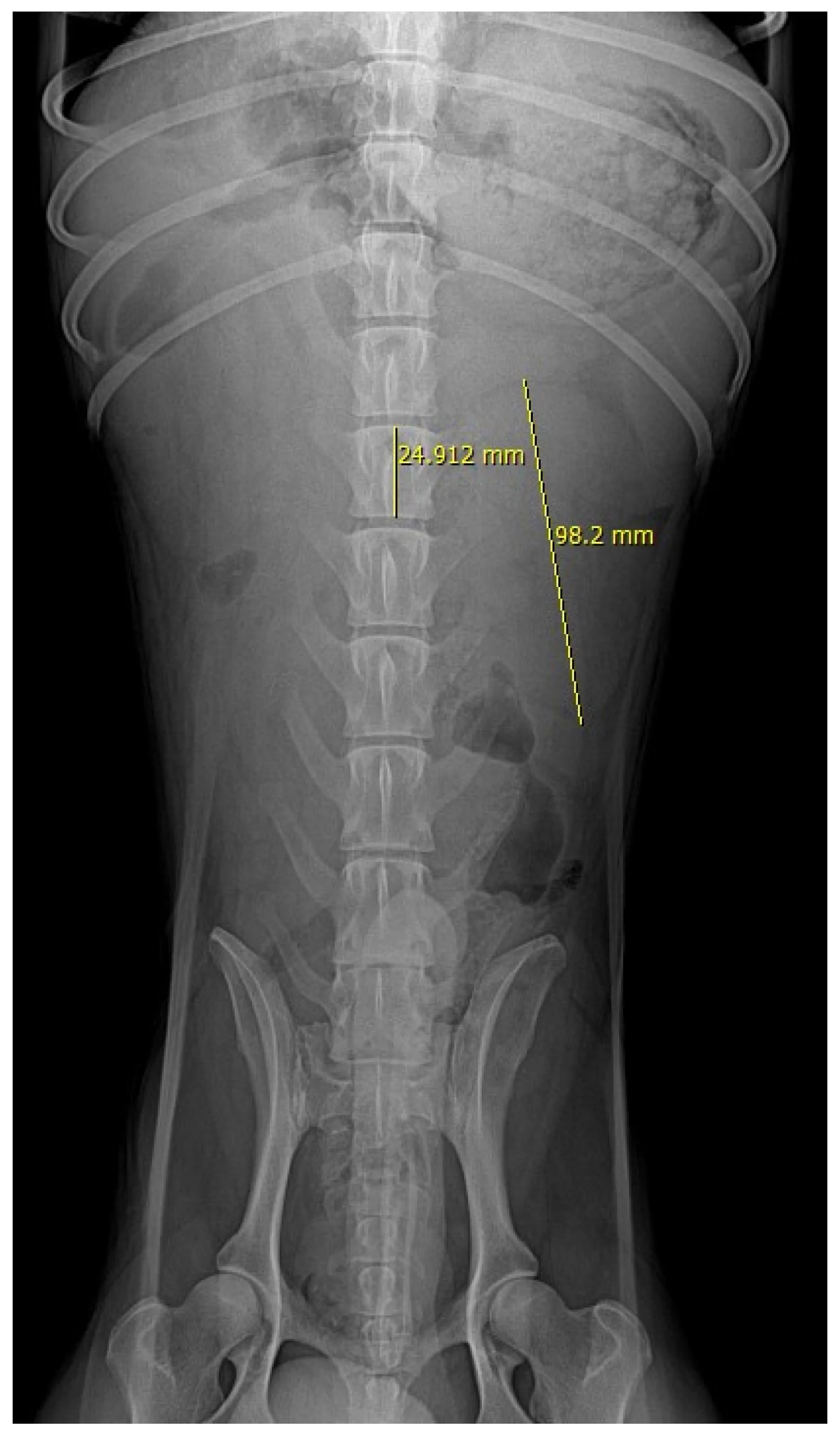
Ventrodorsal abdominal radiograph obtained on presentation, demonstrating renomegaly. The length of the left kidney (98.2 mm) was compared with the length of the second lumbar vertebra (L2; 24.912 mm), yielding a kidney-to-L2 ratio of approximately 3.9, consistent with renal enlargement.

Ventrodorsal abdominal radiography was therefore obtained to corroborate renomegaly (Figure 4). The left kidney measured 98.2 mm, compared with a second lumbar vertebral body (L2) length of 24.912 mm, yielding a kidney-to-L2 ratio of approximately 3.9—exceeding the previously reported mean radiographic ratio in normal adult dogs (2.98 ± 0.44 on ventrodorsal views) [10]—supporting renomegaly consistent with the ultrasonographic findings.

### 2.4. Molecular Diagnosis

A urine sample obtained by cystocentesis on Day 0 was submitted to a commercial veterinary reference laboratory (Green Vet, Yongin, Republic of Korea) and tested using a multiplex real-time PCR panel (GCani™ Urinary Tract Infection-B6 qPCR Detection Kit, Jinslab, Seongnam, Gyeonggi-do, Republic of Korea, co-developed with Green Vet) targeting 19 canine urinary pathogens, following nucleic acid extraction on an automated platform MagNa Pure 96, (Roche, Penzberg, Germany). An internal amplification control, together with a concurrent positive control (Ct 22.66) and negative control (no amplification), confirmed assay validity; testing was performed as a single measurement without technical replicates. On Day 2, the assay was positive for the pathogenic *Leptospira* *LipL32* gene (Ct 31.10; laboratory positive cut-off, Ct < 40), with no other urinary pathogens detected.

This result provides molecular evidence supporting active infection or urinary shedding of pathogenic *Leptospira* spp., but it does not identify the infecting species, serovar, strain, reservoir, or source of exposure. Because testing was performed at an external commercial laboratory, independent data regarding the assay’s analytical sensitivity, specificity, and limit of detection were not available to the authors, and this should be considered when interpreting the reported Ct value.

### 2.5. Treatment

Empirical treatment targeting suspected leptospirosis was initiated on Day 0, based on clinical suspicion, prior to receipt of the PCR result reported on Day 2. Because severe vomiting made oral administration unreliable, intravenous amoxicillin–clavulanate was administered from Day 0 and discontinued once oral doxycycline was initiated on Day 1, following resolution of vomiting; doxycycline was then continued as monotherapy for a total of 14 days, including outpatient administration following discharge. Intravenous fluid therapy with Lactated Ringer’s Solution was calculated from the estimated fluid deficit, based on the upper end of the estimated dehydration range (8% of the Day 0 body weight of 17.3 kg) and rounded to 1440 mL/24 h (60 mL/h) for practical infusion-rate calculation, together with the maintenance requirement (1038 mL/24 h), for an initial combined rate of 6.0 mL/kg/h, subsequently tapered to 4.0 mL/kg/h as hydration status and body weight improved. A summary of pharmacologic and supportive interventions is provided in Table 2.

Throughout hospitalization (Day 0–5), body weight increased progressively (17.3–18.3 kg), blood pressure remained stable (110–120 mmHg, oscillometric), and urine output measured via indwelling urinary catheter remained within the non-oliguric range (2.15–3.0 mL/kg/h, Day 0–4), supporting adequate perfusion and renal function without clinical evidence of fluid overload (Supplementary Table S1). Famotidine and omeprazole were co-administered during the initial period of severe gastrointestinal signs to provide more comprehensive acid suppression, and were continued throughout hospitalization as a precaution against recurrence of gastrointestinal signs despite clinical resolution of vomiting and diarrhea within 24 h. Given the zoonotic potential of leptospirosis, barrier precautions and dedicated isolation housing were used, and the owner was counseled regarding personal medical evaluation and environmental precautions; the owner subsequently consulted a physician, who elected clinical observation without laboratory testing. No other dogs were cohabiting with the patient (Supplementary Note S1).

### 2.6. Outcome and Follow-Up

Vomiting and diarrhea resolved completely within 24 h, and renal and inflammatory biomarkers declined progressively (Table 3, Figure 1 and Figure 2). An isolated, transient increase in c-CRP occurred between Day 2 (54 mg/L) and Day 3 (73 mg/L) despite a concurrent decline in renal biomarkers; the precise cause could not be determined (Supplementary Note S2), and this fluctuation did not affect the overall favorable trend.

By Day 22, the patient was clinically excellent. Creatinine and phosphorus had returned within reference intervals, while BUN and c-CRP were markedly improved but remained mildly above their respective reference intervals. A follow-up urine PCR on Day 22 was negative, consistent with a marked reduction in detectable urinary leptospiral DNA; however, intermittent shedding and bacterial loads below the detection limit cannot be excluded, and this limited follow-up period does not confirm complete long-term renal recovery.

## 3. Discussion

### 3.1. Clinical Significance

Leptospiral infection in unvaccinated dogs and urban environmental exposure are not, in themselves, novel observations. The principal contribution of this report is the detailed, serial clinicopathological documentation of a dog with IRIS Grade V AKI that remained non-oliguric throughout the catheterized period (Day 0–4) and achieved favorable clinical recovery with conventional medical management. Preserved urine output does not, by itself, indicate preserved renal function; however, it is one of several clinical parameters—alongside hemodynamic stability and acid–base status—used to evaluate the indication for EKST [9], rather than serum creatinine alone. This case provides reference data illustrating that severe azotemia does not necessarily indicate oliguric, dialysis-dependent disease, and that non-oliguric status was one relevant factor, among others, considered in this patient’s management.

### 3.2. Diagnostic Interpretation and Limitations of Molecular Testing

A positive urinary *LipL32* real-time PCR provides molecular evidence of infection or shedding involving pathogenic *Leptospira* spp., but it does not identify the infecting serovar, strain, reservoir, or transmission route [11,12]. The microscopic agglutination test (MAT) remains the standard for serovar identification, but its diagnostic utility is limited during the early, hyperacute phase because of the immunological lag phase preceding detectable antibody production [13,14]; MAT, blood PCR, and culture were not performed in this case, limiting further microbiological characterization. Similarly, the negative follow-up PCR on Day 22 is compatible with a reduction in detectable urinary leptospiral DNA but does not establish complete microbiological clearance, given the possibility of intermittent shedding or bacterial loads below the assay’s detection limit [2,13].

### 3.3. Differential Diagnosis

Alternative causes of severe AKI were considered. Ethylene glycol toxicosis was considered unlikely given the absence of a reported exposure history and of calcium oxalate crystalluria, although these findings do not entirely exclude exposure. Pyelonephritis could not be fully excluded, as urine culture was not performed; the absence of bacteriuria on sediment examination reduces but does not eliminate this possibility, and *Leptospira* spp. are not reliably visualized by standard bright-field microscopy. Ischemic or dehydration-associated tubular injury may have contributed but does not fully account for the severity of azotemia or the positive urinary PCR result. Infectious canine hepatitis was considered unlikely given normal hepatic enzyme activities (ALT 94 U/L, ref. 10–102; AST 35 U/L, ref. 0–48) and the absence of ultrasonographic findings suggestive of hepatitis or biliary tract involvement, and tick-borne diseases (babesiosis, ehrlichiosis, anaplasmosis) were considered less likely given a normal platelet count and the patient’s predominantly urban lifestyle, although comprehensive testing for these conditions was not performed.

### 3.4. Therapeutic Rationale

Antimicrobial and fluid therapy in this patient followed existing consensus recommendations rather than representing a novel protocol [2,13]. Amoxicillin–clavulanate was selected as the initial parenteral agent because beta-lactams are recommended during the acute leptospiremic phase, with subsequent transition to doxycycline—the standard agent for eliminating renal colonization—once oral administration became feasible [2,13]. The decision not to pursue EKST was based on the patient’s non-oliguric status—considered alongside, and not in place of, hemodynamic stability, acid–base status, and progressive clinical improvement—rather than on serum creatinine alone [9]. Non-oliguric status alone does not equate to preserved renal function; the favorable outcome in this patient reflects the combination of these clinical parameters rather than urine output in isolation. This favorable outcome should be interpreted as the result observed in this individual patient rather than evidence that conventional management is generally sufficient for dogs with IRIS Grade V AKI.

### 3.5. Epidemiological Considerations

The dog’s history of exposure to urban stagnant water represents a plausible, but not confirmed, source of infection. Urban leptospirosis outbreaks in companion dogs have been reported internationally. In a 2021 outbreak among client-owned dogs in Los Angeles County, USA, a survey of self-selected respondents (n = 102) found that 77% were unaware of the clinical signs of leptospirosis or that the disease is zoonotic [6]. In a 2017 outbreak among 11 dogs in Osaka Prefecture, Japan, in which 9 dogs died, walking along a shared riverbed was identified as a significant risk factor, and the suspected causative serovar (Australis) had no licensed canine vaccine available in Japan at that time [7]—illustrating that regional vaccine availability, rather than vaccination status alone, can influence population-level susceptibility. Neither report establishes transmission dynamics, population-level risk, or outbreak potential specific to the Republic of Korea. A recent Korean serosurvey found no detectable *Leptospira* antibodies among sampled stray dogs [15]. Whether this finding extends to owned companion dogs, such as the patient in this report, remains untested; indeed, a separate serosurvey identified leptospiral antibodies specifically in domestic companion dogs in South Korea [5], suggesting that immune status may differ meaningfully between these two populations. The immunological naivety of urban companion dogs to *Leptospira* should therefore be regarded as an untested hypothesis rather than an established finding, pending serosurveys conducted directly in owned dog populations.

### 3.6. Limitations

This report has several limitations. As the PCR assay was performed at an external commercial laboratory, independent data regarding its analytical validation were not available to the authors. MAT serology, blood PCR, and urine and blood culture were not performed, precluding serovar identification and definitive microbiological confirmation. Persistent, low-level urinary shedding by clinically recovered or asymptomatic carrier dogs has been documented as an ongoing public health concern [16], underscoring the importance of the long-term monitoring noted above. Electrolyte and blood gas parameters were measured only at presentation (Day 0) and were not repeated during hospitalization; consequently, their trajectory over the course of treatment could not be documented. However, the absence of hypotension, the progressive improvement in renal and inflammatory biomarkers, and the steady increase in body weight throughout hospitalization (Supplementary Table S1) are consistent with the absence of major, clinically undetected electrolyte or acid–base derangement during this period. No renal histopathology was available to confirm the extent of tubular injury or the absence of interstitial fibrosis. Advanced ultrasonographic modalities (e.g., Doppler, contrast-enhanced ultrasonography, elastography) were not performed. Follow-up was limited to Day 22; normalization of serum creatinine at this time point does not confirm complete long-term renal recovery.

## 4. Conclusions

This case documents a dog with non-oliguric IRIS Grade V AKI and molecular evidence supporting suspected leptospirosis that achieved favorable clinical recovery, with progressive resolution of clinical signs and biochemical improvement, with conventional medical management, without extracorporeal kidney support. These findings should be interpreted as a single-patient clinical course rather than evidence that this outcome is generalizable to all dogs with severe AKI, that urban environmental exposure was confirmed as the source of infection, or that early PCR testing was directly responsible for recovery. Clinicians should consider leptospirosis in the differential diagnosis of AKI in urban dogs, use urine output alongside serum creatinine when evaluating the need for extracorporeal support, and continue to emphasize preventive vaccination, noting that vaccine composition and regional serovar coverage may vary.

## Figures and Tables

**Table 1:** Baseline (Day 0) hematological, biochemical, blood gas, and urinalysis findings.

Parameter	Reference Range	Result
Hematology		
WBC (K/µL)	5.05–16.76	17.64
RBC (M/µL)	5.65–8.87	7.44
Hemoglobin (g/dL)	13.1–20.5	19.6
Platelet (K/µL)	148–484	248
Reticulocytes, absolute (K/µL)	10–110	5.9
Reticulocytes (%)	—	0.1
MCV (fL)	—	66.3
MCHC (g/dL)	—	34.9
**Serum Biochemistry**		
AST (U/L)	0–48	35
ALT (U/L)	10–102	94
Albumin (g/dL)	2.3–4.0	2.8
Total protein (g/dL)	5.2–8.2	6.4
Glucose (mg/dL)	70–143	107
**Blood gas & Electrolytes**		
Sodium (mmol/L)	139–151	154
Potassium (mmol/L)	3.6–5.3	3.1
Chloride (mmol/L)	107–122	109
Anion gap (mmol/L)	7–16	25
Blood pH (venous)	—	7.47
Bicarbonate (mmol/L)	21–28	24.1
pCO_2_ (mmHg)	27.0–50.0	36.0
Base excess (mmol/L)	—	−0.4
Lactate (mmol/L)	—	1.2
**Urinalysis**		
Urine specific gravity	1.008–1.030 *	1.015
Urine pH	—	6.5
Urine protein-to-creatinine ratio	0.2–0.4	0.79
Urine glucose (mg/dL)	0–49	100

* Population reference interval; canine urine specific gravity should be interpreted relative to hydration and renal function.

**Table 2:** Summary of pharmacologic and supportive interventions.

Agent/Intervention	Dose/Rate	Route	Frequency	Duration/Notes
Amoxicillin–clavulanate	12.5 mg/kg	IV	BID	Day 0–1; discontinued at transition to doxycycline
Doxycycline	5 mg/kg	PO	BID	14 days total (from Day 1, including outpatient course)
Lactated Ringer’s Solution	6.0 → 4.0 mL/kg/h	IV	Continuous	Deficit 1440 mL/24 h + maintenance 1038 mL/24 h; tapered as hydration improved
Maropitant citrate	1 mg/kg	IV	SID	4 days (antiemetic)
Famotidine	0.5 mg/kg	IV	BID	Throughout hospitalization
Omeprazole	0.5 mg/kg	PO	SID	Throughout hospitalization
Butorphanol	0.2 mg/kg	IV	BID	First 3 days (analgesia)
Sevelamer	45 mg/kg	PO	BID	With food, for hyperphosphatemia; Throughout hospitalization

**Table 3:** Temporal changes in major clinicopathological parameters from admission (Day 0–5) to follow-up (Day 22).

Parameter	Reference Range	Day 0	Day 1	Day 2	Day 3	Day 4	Day 5	Day 22
**BUN** **(mg/dL)**	6–25	175.8	145.8	127.6	91.8	70.1	53.7	26.7
**Creatinine** **(mg/dL)**	0.4–1.8	11.1	9.29	8.01	7.07	6.47	3.04	1.78
**Phosphorus** **(mg/dL)**	2.5–6.8	13.6	9.9	8	7.2	6.1	5.9	5.5
**c-CRP** **(mg/L)**	0–9	95	66	54	73	45	28	14
**Hematocrit** **(%)**	37.3–61.7	46	35.5	35.8	—	—	—	—

## Data Availability

The data presented in this study are available on request from the corresponding author because the presence of information that could compromise the privacy of the animal’s owner within the original clinical records. De-identified imaging data, laboratory findings, treatment protocols, and follow-up data are available from the corresponding author upon reasonable request.

## Supplementary Materials

The following supporting information can be downloaded at: https://www.mdpi.com/article/doi/s1: **Table S1: Daily body weight, blood pressure, and urine output (Day 0–5); Note S1: Extended description of supportive care, infection control, and owner counseling; Note S2: Extended discussion of the transient c-CRP fluctuation (Day 2–3).**

## Abbreviations

The following abbreviations are used in this manuscript:

AKI	Acute kidney injury
IRIS	International Renal Interest Society
BUN	Blood urea nitrogen
PCR	Polymerase chain reaction
c-CRP	Canine C-reactive protein
MAT	Microscopic agglutination test
EKST	Extracorporeal kidney support therapy
DHPP	Distemper, hepatitis, parvovirus, parainfluenza (vaccine)
ALT	Alanine aminotransferase
AST	Aspartate aminotransferase
MCV	Mean corpuscular volume
MCHC	Mean corpuscular hemoglobin concentration
IV	Intravenous
PO	Per os (oral)
BID	Bis in die (twice daily)
SID	Semel in die (once daily)
