# Supplementary material for: Non-Oliguric IRIS Grade V Acute Kidney Injury Associated with Suspected Leptospirosis in an Urban Dog Unvaccinated Against Leptospirosis from the Republic of Korea: A Case Report
Source: Vet Sci. 2026 Sep 17;13(9):982. doi: 10.3390/vetsci13090982 (PMC13612007; doi:10.3390/vetsci13090982)
Supplement: Supplementary file 1 [file vetsci-13-00982-s001.zip › vetsci-4554499-supplementary.pdf]

# Supplementary Materials

*Non-Oliguric IRIS Grade V Acute Kidney Injury Associated with Suspected Leptospirosis in an Unvaccinated Urban Dog from the Republic of Korea: A Case Report*

Taehyun Roh and Kunho Song

## Table S1. Daily Body Weight, Blood Pressure, and Urine Output (Day 0–5)

| Parameter | Day 0 | Day 1 | Day 2 | Day 3 | Day 4 | Day 5 |
|---|---|---|---|---|---|---|
| Body weight (kg) | 17.3 | not recorded | 17.9 | 18.1 | 18.2 | 18.3 |
| Blood pressure (mmHg, oscillometric) | 110–120 | 110–120 | 110–120 | 110–120 | 110–120 | 110–120 |
| Urine output (mL/kg/h, indwelling catheter) | 2.15 | 2.7 | 3.0 | 2.2 | 2.3 | catheter removed |

Body weight increased progressively over the hospitalization period, consistent with adequate fluid balance without clinical evidence of fluid overload **(no respiratory distress, tachypnea, or pulmonary crackles were observed at any point).** Blood pressure remained within a stable range throughout hospitalization. Urine output, measured via an indwelling urinary catheter placed on Day 0 and removed after Day 4, remained within the non-oliguric range (>1 mL/kg/h) at every time point, supporting the non-oliguric classification of this patient's acute kidney injury referenced in the main text.

## Note S1. Extended Description of Supportive Care, Infection Control, and Owner Counseling

In addition to the pharmacologic interventions summarized in Table 3 of the main text, the patient maintained a spontaneous appetite throughout hospitalization, and assisted or syringe feeding was not required at any point. No additional potassium supplementation beyond that provided by Lactated Ringer's Solution was administered, as the Day 0 hypokalemia (3.1 mmol/L) was considered mild and **unlikely to require independent correction.**

Given the zoonotic potential of leptospirosis, hospital staff used barrier precautions, including masks, protective goggles, and gloves, when handling the patient or its urine. The patient was housed in a dedicated isolation area with separate handling and disposal of waste materials throughout hospitalization. The owner was counseled regarding the zoonotic risk associated with leptospirosis and was advised to seek personal medical evaluation; the owner subsequently consulted a physician, who **opted for clinical observation of any emerging symptoms rather than** laboratory testing. No other dogs were cohabiting with the patient at the time of diagnosis or during the follow-up period. Following clinical recovery, no additional leptospirosis vaccination was documented during the study period; the owner was counseled on environmental precautions (e.g., avoiding stagnant water sources) and on the importance of future leptospirosis vaccination, although subsequent vaccination status could not be confirmed by the authors.

## Note S2. Extended Discussion of the Transient c-CRP Fluctuation (Day 2–3)

As noted in the main text (Section 2.6), c-CRP transiently increased from 54 mg/L on Day 2 to 73 mg/L on Day 3, despite a concurrent and consistent decline in renal biomarkers (BUN and creatinine) across the same interval (Table 1).

**Several factors could theoretically contribute to this isolated fluctuation, including the distinct kinetics of hepatic c-CRP synthesis and clearance relative to renal biomarker resolution, a transient response to the Day 1 antimicrobial transition, or the indwelling urinary catheter; however, none of these possibilities were specifically investigated, and the precise cause could not be determined from the available data.**

Because c-CRP was not measured more frequently than once daily, and because no additional diagnostic testing (e.g., repeat urinalysis, blood culture) was performed to investigate this fluctuation further, no additional characterization was possible. Importantly, this single-day fluctuation did not alter the overall favorable downward trend in either renal or inflammatory biomarkers, and c-CRP had declined to within a mildly elevated range (14 mg/L; reference interval, 0–9 mg/L) by Day 22.
